# Supplementary material for: Limited intestinal inflammation despite diarrhea, fecal viral RNA and SARS-CoV-2-specific IgA in patients with acute COVID-19
Source: Sci Rep. 2021 Jun 25;11:13308. doi: 10.1038/s41598-021-92740-9 (PMC8233421; doi:10.1038/s41598-021-92740-9)
Supplement: Supplementary file 2 — Supplementary Information 2. [file 41598_2021_92740_MOESM2_ESM.pdf]

Limited intestinal inflammation despite diarrhea, fecal viral RNA and SARS-CoV-2-specific IgA in patients with acute COVID-19

Graham J. Britton, Alice Chen-Liaw, Francesca Cossarini, Alexandra E. Livanos, Matthew P. Spindler, Tamar Plitt, Joseph Eggers, Ilaria Mogno, Ana S. Gonzalez-Reiche, Sophia Siu, Michael Tankelevich, Lauren Tal Grinspan, Rebekah E. Dixon, Divya Jha, Adriana van de Guchte, Zenab Khan, Gustavo Martinez-Delgado, Fatima Amanat, Daisy A. Hoaglan, Benjamin R. tenOever, Marla C. Dubinsky, Miriam Merad, Harm van Bakel, Florian Krammer, Gerold Bongers, Saurabh Mehandru and Jeremiah J. Faith

Supplementary Materials:

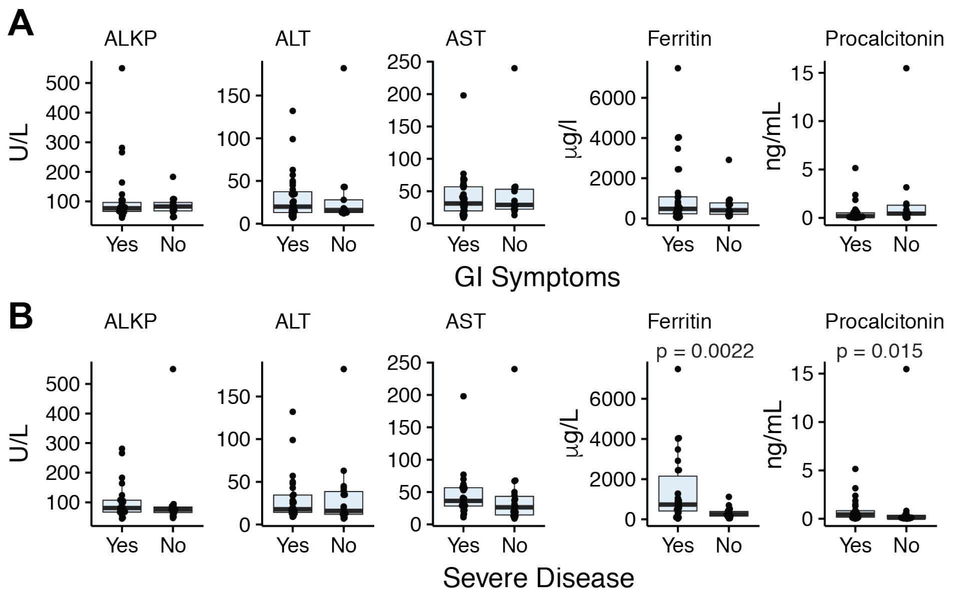

Fig. S1. Gastrointestinal symptoms and serologic parameters in hospitalized COVID-19 patients. **(A)** Liver enzymes and laboratory values in hospitalized COVID-19 patients with and without GI symptoms. **(B)** Liver enzymes and laboratory values in hospitalized COVID-19 patients with severe disease or non-severe disease. Each point represents an individual value for a patient, the box plot shows the median and the interquartile range and the p-values are calculated using the Mann-Whitney test with significance defined as $p<0.05$.

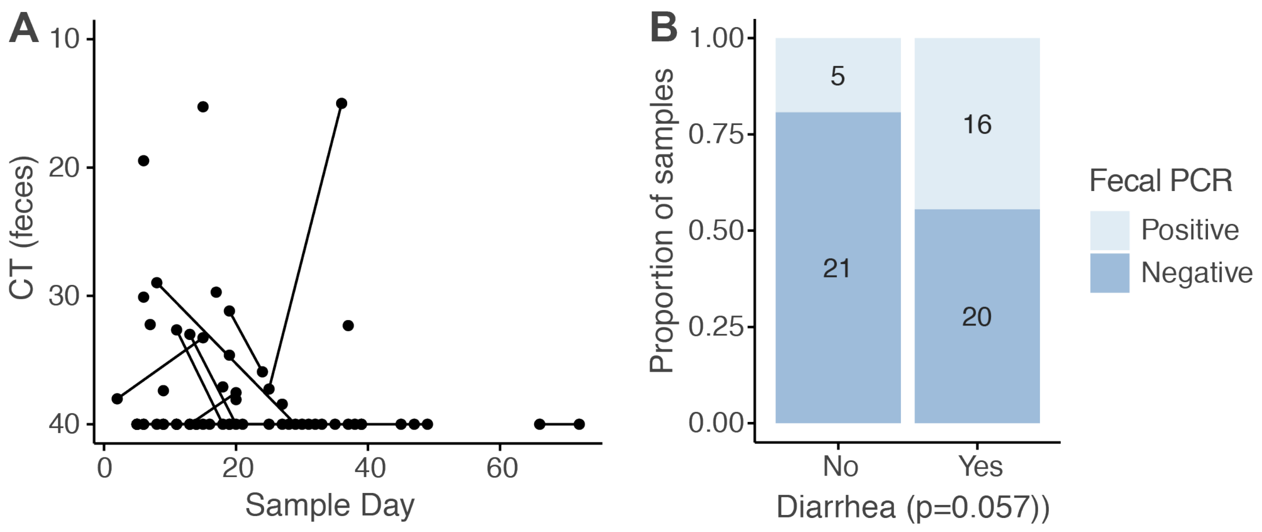

Figure S2. Detection of SARS-CoV-2 virus genome in stool. (A). Each point shows the median SARS-CoV-2 Ct from one sample, plotted against the sampling day relative to the onset of symptoms. Lines connect samples from the same donor. (**B**) The proportion of positive and negative fecal SARS-CoV-2 PCR results from donors with and without diarrhea. p value - Fisher's exact test.

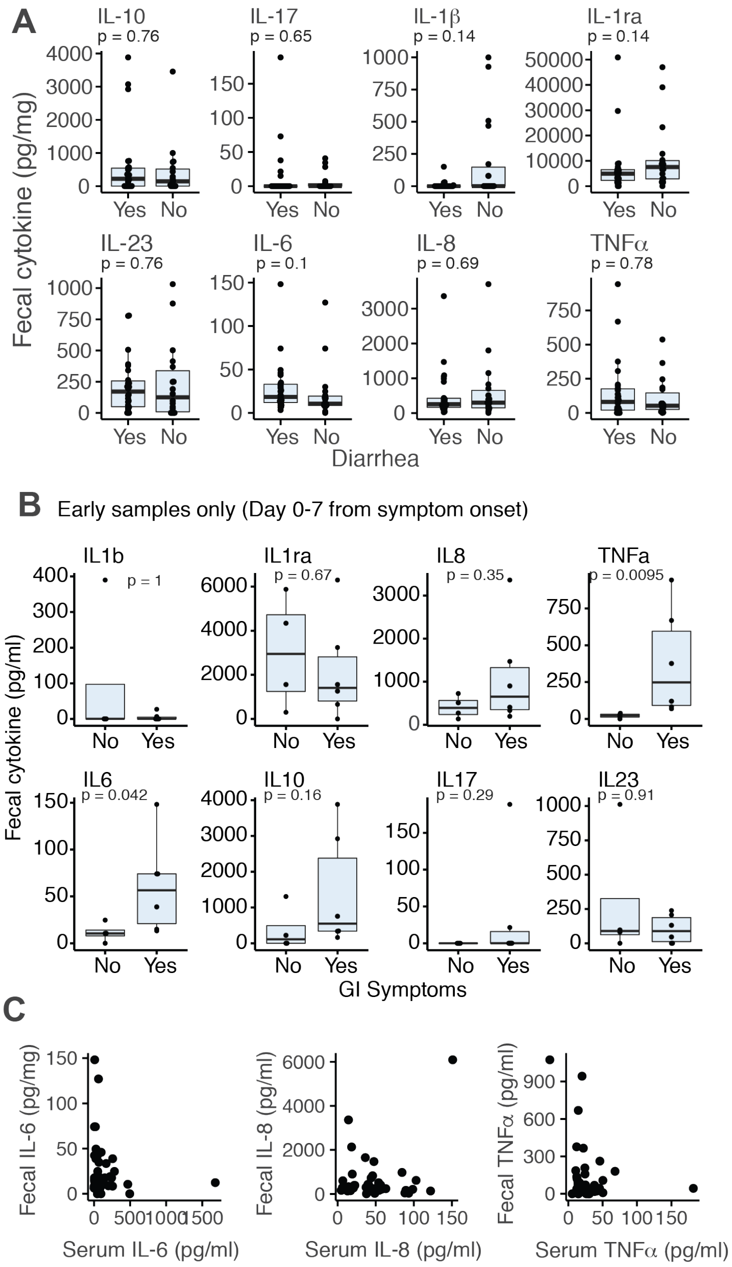

Fig. S3. Fecal cytokine levels in COVID-19 patients. (**A**) Concentrations of the indicated cytokine in fecal samples from COVID-19 patients with and without diarrhea. P values - Mann-Whitney. **(B)** Concentrations of the indicated cytokine in fecal samples collected within 7 days of symptom onset from COVID-19 patients with and without GI symptoms. P values - Mann-Whitney. **(C)** Relationship between concentrations of the indicated cytokine in feces and serum.

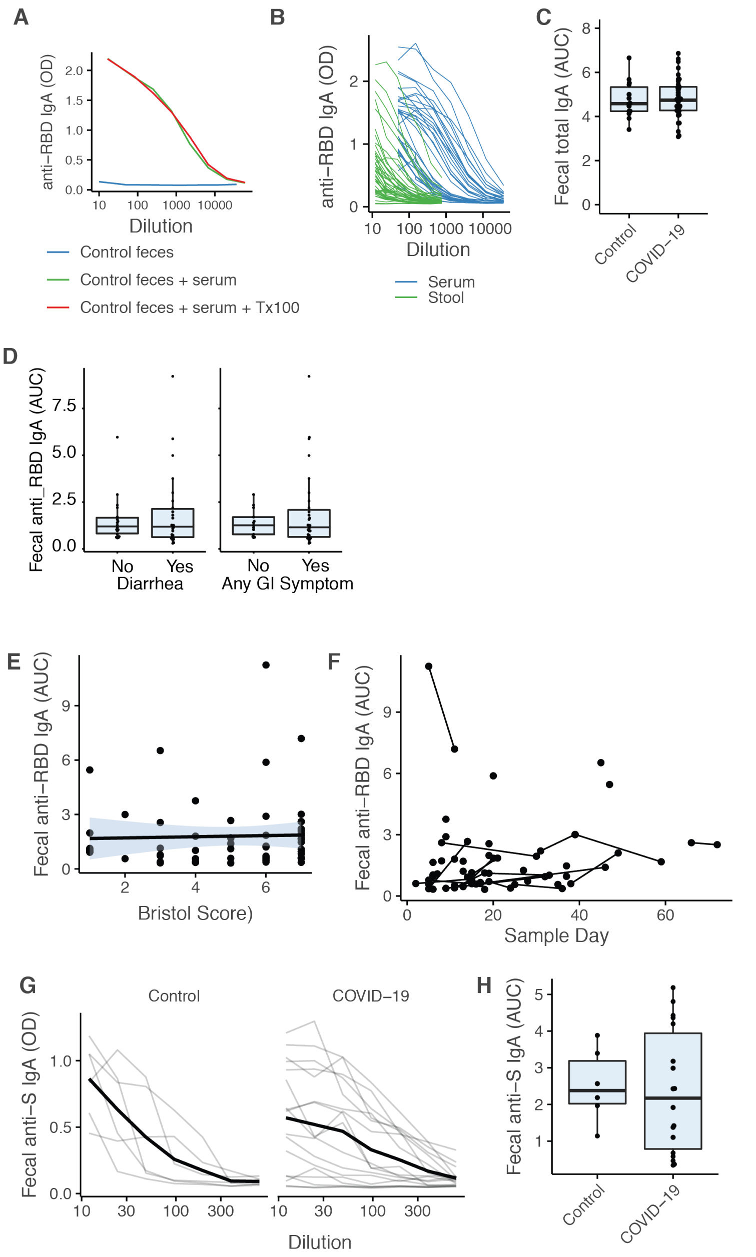

Figure. S4. SARS-CoV-2-specific IgA in stool of COVID-19 patients. (A) Triton X-100 does not reduce detection of anti-RBD IgA in a serum sample from a seropositive donor serum spiked into a healthy donor stool sample. **(B)** Relative titers of serum and fecal anti-RBD IgA in samples from COVID-19 patients. **(C)** Relative titers of total IgA in feces of control donors and COVID-19 patients. (**D** and **E)** Anti-SARS-CoV-2 RBD specific IgA titers are not different in donors with diarrhea or donors reporting any GI symptom. (D – averaged by donor, E - by sample). **(F)** Anti-SARS-CoV-2 RBD IgA in feces over time relative to reported symptom onset. Each point shows data from one sample and lines connect samples from the same donor. (**G**) Anti-SARS-CoV-2 Spike protein IgA in control donors and COVID-19 patients. (**H**) Relative titers of anti-SARS-CoV-2 Spike protein IgA in control donors and COVID-19 patients.

A

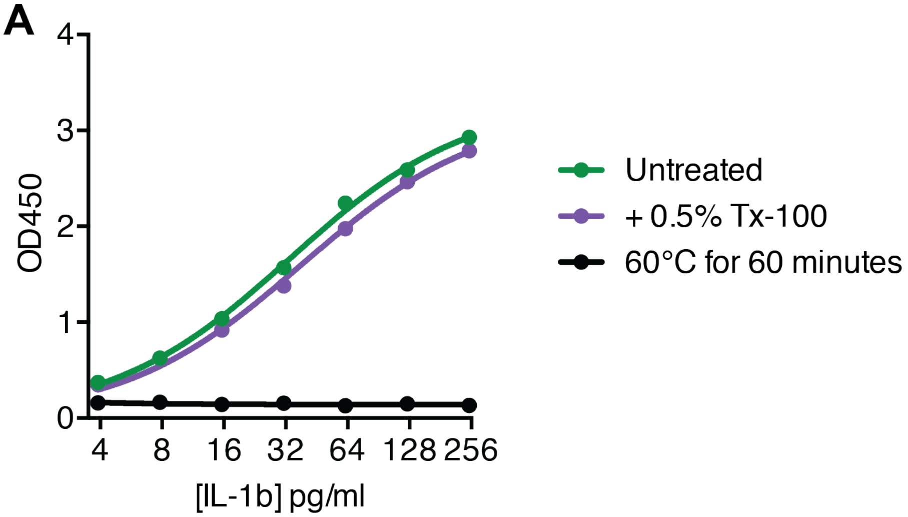

Figure S5. Virus inactivation method compatible with cytokine ELISAs. (A) Recombinant human IL-1β was spiked into a stool homogenate from a healthy donor and either heated at 60°C for 60 minutes or treated with 0.5% Triton X-100 (Tx-100) for 60 minutes at 4°C before ELISA analysis.

Table S1: Sample collection and description

The number of each sample type and the day of collection (median +/- the range), relative to the onset of COVID-19 symptoms.

	1st stool sample	2nd stool sample	Serum
Number of samples	44	18	32
Median time in days (range) from symptom onset to sample collection	16 (2-66)	24.5 (11-72)	16 (3-46)

Table S2: COVID-19 treatments in patients with and without GI symptoms
The absolute number and percentage of the cohort (in parentheses) who received the indicated therapeutic. Statistical comparisons are by Fisher's exact test.

	Total (n=44)	GI symptoms (n=31)	No GI symptoms (n=13)	p-value
Antibiotics	28 (63.6)	17 (54.8)	11 (84.6)	0.09
Azithromycin	17 (38.6)	8 (25.8)	9 (69.2)	0.02
Vancomycin	13 (29.5)	7 (22.6)	6 (46.2)	0.16
Ceftriaxone (CTX)	17 (38.6)	9 (29.0)	8 (61.5)	0.09
Vancomycin + CTX	8 (18.2)	4 (12.9)	4 (30.8)	0.21
Cefepime	12 (27.3)	8 (25.8)	4 (30.8)	0.73
Other antibiotics	10 (22.7)	5 (16.1)	5 (38.5)	0.13
Plasma	20 (45.5)	13 (41.9)	7 (53.8)	0.52
Plasma before sample collection	19 (43.2)	13 (41.9)	6 (46.2)	>0.99
Hydroxychloroquine	19 (43.2)	12 (38.7)	7 (53.8)	0.51
Remdesivir	13 (29.5)	8 (25.8)	5 (38.5)	0.48
Steroids	18 (40.9)	13 (41.9)	5 (38.5)	>0.99
Therapeutic anticoagulation	31 (70.5)	22 (71.0)	9 (69.2)	>0.99

Table S3: Disease severity associated with composite outcome of ICU admission or death
The absolute number and percentage of the cohort (in parentheses) with each indicated COIVD-19 severity. Statistical comparisons are by Fisher's exact test.

**Severity on admission**	ICU admission or death (n=14)	Neither (n=30)	p-value
Mild or moderate	6 (42.9)	22 (73.3)	
Severe	8 (57.1)	8 (26.7)	0.09
**Peak severity**			
Mild or moderate	2 (14.3)	16 (53.3)	
Severe	12 (85.7)	14 (46.7)	**0.02**

Table S4. PCR primer sequences		
**Primer**	**Sequence (5'-3')**	**Reference**
2019-nCoV_N1 Forward	GACCCCAAAATCAGCGAAAT	[57]
2019-nCoV_N1 Reverse	TCTGGTTACTGCCAGTTGAATCTG	[57]
2019-nCoV_N1 Probe	ACCCCGCATTACGTTTGGTGGACC	[57]
2019-nCoV_N2 Forward	TTACAAACATTGGCCGCAAA	[57]
2019-nCoV_N2 Reverse	GCGCGACATTCCGAAGAA	[57]
2019-nCoV_N2 Probe	ACAATTTGCCCCCAGCGCTTCAG	[57]
RNase P Forward	AGATTTGGACCTGCGAGCG	[57]
RNase P Reverse	GAGCGGCTGTCTCCACAAGT	[57]
RNase P Probe	TTCTGACCTGAAGGCTCTGCGCG	[57]
Nsp14 Forward	TGGGGYTTTACRGGTAACCT	[45]
Nsp14 Reverse	AACRCGCTTAACAAAGCACTC	[45]
Hypoxanthine phosphoribosyl transferase (HPRT) Forward	CAACAGGCTTTTCCTGGTT	[58]
Hypoxanthine phosphoribosyl transferase (HPRT) Reverse	GGCTACTCTGCCCATGAAGA	[58]
ß-actin Forward	CCCAGCACAATGAAGATCAA	[59]
ß-actin Reverse	ACATCTGCTGGAAGGTGGAC	[59]
Glyceraldehyde 3-phosphate dehydrogenase (GAPDH) Forward	GTCGTGGAGTCTACTGGTGTCTTC	[60]
Glyceraldehyde 3-phosphate dehydrogenase (GAPDH) Reverse	GTCATATTTCTCGTGGTTCACACC	[60]
16S 8F (qPCR control)	AGAGTTTGATCCTGGCTCAG	[61]
16S 1391R (qPCR control)	GACGGGCGGTGWGTRCA	[61]

Supplemental Text:

Below is the clinical questionnaire administered to all recruited COVID-19 patients.

Clinical Questionnaire:

Simple questionnaire:

1. Diarrhea (Y/N)?
2. Nausea (Y/N)?
3. Vomiting (Y/N)?

Detailed questionnaire:

1. Are you having diarrhea? (Y/N)
2. When did your diarrhea start? (date)
3. When did any of your other symptoms start including fever or other symptoms (cough, shortness of breath, sore throat? (date)
4. Did your diarrhea end or is it ongoing?
5. How many stools are you having per day?
6. Is there any bood in your stool? (Y/N)
7. Are stools large volume and mostly water? (Y/N)
8. Are you waking up in the middle of the night to have bowel movement? (Y/N)
9. Do you have abdominal pain? (Y/N)

Other GI symptoms / history question:

1. Is your appetite decreased? (Y/N)
2. Are you having nausea? (Y/N)
3. Are you having any episodes of vomiting? (Y/N)
